# Adherence to a healthy and potentially sustainable Nordic diet is associated with child development in The Norwegian Mother, Father and Child Cohort Study (MoBa)

**DOI:** 10.1186/s12937-022-00799-5

**Published:** 2022-07-18

**Authors:** Kristine Vejrup, Neha Agnihotri, Elling Bere, Synnve Schjølberg, Marissa LeBlanc, Elisabet Rudjord Hillesund, Nina Cecilie Øverby

**Affiliations:** 1 Department of Nutrition and Public Health, Centre for Lifecourse Nutrition, University of Agder, Kristiansand, Norway; 2 Norwegian Armed Forces Medical Services, Sessvollmoen, Norway; 3 Department of Sport Science and Physical Education, University of Agder, Kristiansand, Norway; 4 Department of Health and Inequality, Norwegian Institute of Public Health, Oslo, Norway; 5 Centre for Evaluation of Public Health Measures, Norwegian Institute of Public Health, Oslo, Norway; 6 Department of Child Health and Development, Norwegian Institute of Public Health, Oslo, Norway; 7 Oslo Centre for Biostatistics and Epidemiology, Oslo University Hospital, Oslo, Norway

**Keywords:** New Nordic diet, Nordic diet, Child diet, Healthy diet score, Child development, MoBa, MBRN

## Abstract

**Background:**

The rapid neurodevelopment that occurs during the first years of life hinges on adequate nutrition throughout fetal life and early childhood. Therefore, adhering to a dietary pattern based on healthy foods during pregnancy and the first years of life may be beneficial for future development. The aim of this paper was to investigate the relationship between adherence to a healthy and potentially sustainable Nordic diet during pregnancy and in early childhood and child development.

**Methods:**

This study is based on the Norwegian Mother, Father and Child Cohort Study (MoBa) and uses data from the Medical Birth Registry of Norway (MBRN). In 83,800 mother-child pairs, maternal pregnancy diet and child diet at 6 months, 18 months and 3 years were scored according to adherence to the New Nordic Diet (NND). NND scores were calculated both as a total score and categorized into low, medium, or high adherence. Child communication and motor development skills were reported by parents at 6 months, 18 months, 3 and 5 years, using short forms of the Ages and Stages Questionnaire and the Child Development Inventory. Associations of NND adherence with child development were estimated with linear and logistic regression in crude and adjusted models.

**Results:**

When examining the NND and child developmental scores as percentages of the total scores, we found positive associations between the NND scores (both maternal pregnancy diet and child diet) and higher scoring on child development (adjusted $\hat{\beta}$ s [95% confidence intervals] ranging from 0.007 [0.004, 0.009] to 0.045 [0.040, 0.050]). We further found that low and medium adherence to NND were associated with higher odds of later emerging developmental skills compared to high NND adherence at nearly all measured timepoints (odds ratios [95% CI] ranging from significant values 1.15 [1.03–1.29] to 1.79 [1.55, 2.06] in adjusted analyses).

**Conclusions:**

Our findings support that adherence to a healthy and potentially sustainable diet early in life is important for child development every step of the way from pregnancy until age 5 years.

**Supplementary Information:**

The online version contains supplementary material available at 10.1186/s12937-022-00799-5.

## Background

The rapid neurodevelopment that occurs during fetal life and the first years after birth represents a particularly vulnerable phase nutritionally [1]. Insufficient intake of micronutrients such as folic acid, choline, omega-3 polyunsaturated fatty acids, vitamin B12, zinc, iron, and iodine during pregnancy and/or early infancy, have shown to be associated with impaired neurocognitive development in children [2, 3]. In addition to providing specific nutrients, healthy dietary patterns have been suggested to promote a healthy neurocognitive development through changes in cellular processes, neuroplasticity, or epigenetic mechanisms [4, 5]. There are also indications that an unhealthy diet could limit or delay typical development [3, 6]. The current literature increasingly focuses on the first 1000 days of life as a critical developmental period [1]. Maternal diet quality during pregnancy has previously been shown to be positively associated with child communication and motor development [7] and a causal association between breastfeeding and child cognition and intelligence has also been established [8]. A small, but positive association between a healthy dietary pattern during infancy and early childhood and subsequent child developmental outcomes and/or a negative association between “unhealthy” patterns and cognitive measures has been shown in a systematic review from 2016 [4]. However, as some parts of the brain continue to develop throughout childhood and adolescence [3], a deeper understanding of the overall role and impact of longer-term exposure of potentially healthy dietary patterns on child development is needed.

The health benefits of a Nordic diet have increasingly become an area of interest, as there is an increasing focus on regional and environment-friendly diets [9–12]. The theoretically defined concept of the New Nordic Diet (NND) encompasses foods that are locally available and traditionally consumed in the Nordic countries, and additionally considers the sustainability potential of the diet [9–11]. Previous studies have shown that the Nordic diet, in various forms, is associated with several health-related outcomes in adults [13–20], but the literature regarding children remains scarce. The Danish OPUS-study examined health-effects of offering school meals based on the Nordic diet to school children aged 8–11 years over 6 months [21], and the intervention improved reading comprehension compared to controls, but not concentration performance [22]. Being rich in healthy foods such as fish, oats, whole grain, and root vegetables, it is plausible that adhering to a healthy Nordic diet from pregnancy throughout early childhood could affect child development positively, with immediate as well as long-term impact [11, 23].

Maternal and child diet scores aiming to capture a potentially healthy, local, and traditional Nordic dietary pattern based on the NND have previously been developed in the Norwegian Mother, Father and Child Cohort Study (MoBa) [18, 24]. In the current study, we aim to examine associations between adherence to a healthy and potentially sustainable Nordic diet during pregnancy and in early childhood and measures of child communication and motor development at different ages in preschool years (6 months, 18 months, 3 years and 5 years of age).

## Material and methods

### Study population

This study was conducted within MoBa, which is a population-based pregnancy cohort study conducted by the Norwegian Institute of Public Health [25]. Pregnant women were recruited from all over Norway from 1999 to 2008, and 41% of those invited consented to participate. The cohort now includes 114.500 children, 95.200 mothers and 75.200 fathers. The study also includes data from the Medical Birth Registry Norway (MBRN), a national health registry containing information about all births in Norway [26]. The two datasets are linked by using the Norwegian National security number which is available in all Norwegian National health registries. The linkage is performed by the MoBa data team and the research file contains an anonymous serial number. The MoBa cohort study is an ongoing longitudinal health study where data still is collected from participants. The current study is based on MoBa version 12 of the quality-assured data files released for research in January 2019. Response rate for the questionnaires answered during pregnancy (Q1-Q3) was between 91 and 95% with decreasing participation rate over time. When the child was 3 years and 5 years old the response rate was at 59 and 54% respectively [25].

**Fig. 1:**
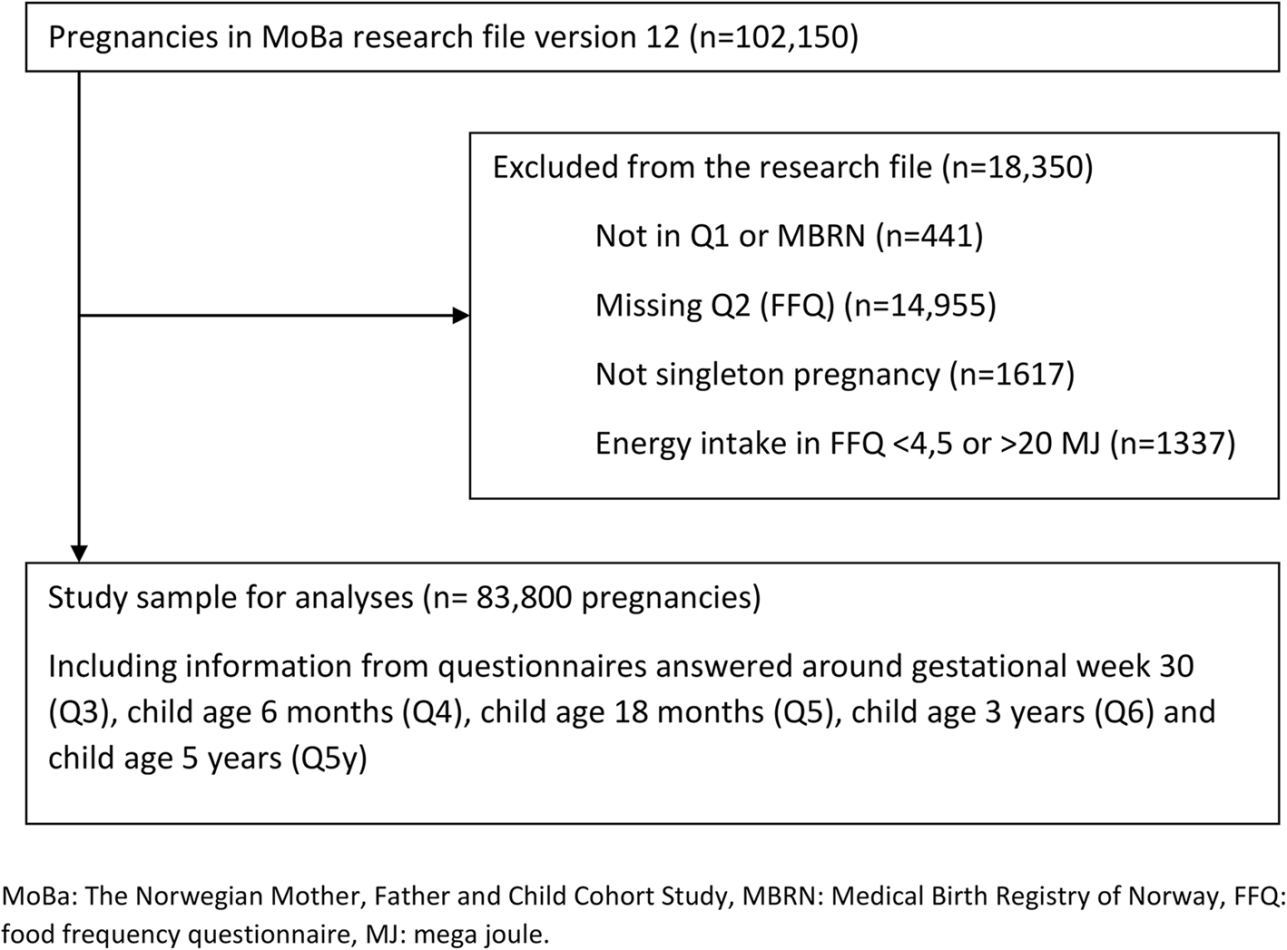
Flow chart of inclusion of participants in the study

We included participants who had responded to the baseline questionnaire (Q1) around gestational week 17, covering general health and sociodemographic information, the food frequency questionnaire (FFQ) (Q2) answered around gestational week 22 and participants who were registered in the MBRN with singleton births. We excluded women with calculated energy intakes outside the range 4.5–20 MJ/day. The final study population consisted of a baseline 83,800 mother-child pairs (Fig. 1).

### Ethics

The establishment of MoBa and initial data collection was based on a license from the Norwegian Data Protection Agency and approval from The Regional Committees for Medical and Health Research Ethics. All MoBa participants provided written informed consent before enrolment into the study. The MoBa cohort is now based on regulations related to the Norwegian Health Registry Act. The current study was approved by The Regional Committees for Medical and Health Research Ethics (2019/339).

### Main exposure

NND scores for maternal pregnancy diet and child diet at 6 and 18 months and 3 years have been developed and are described in detail in previously published papers [18, 24, 27]. A brief summary from these papers is presented here.

The child NND scores were developed under the rationale of being as similar as possible to a previously developed maternal NND score in MoBa [18]. Despite referring to the child diet scores as ‘child NND scores’ in this paper for simplicity reasons, it should be noted that they may not reflect the NND to the same extent as the maternal NND score. The NND scores and their corresponding subscales are presented in Table 1.

**Table 1:** Description of the New Nordic Diet scores

Maternal score	6-months score	18-months score	3-years score
**Scoring range:** 0–10 **Categories:** Low 0–3, medium 4–5, high 6–10	**Scoring range:** 0–6 **Categories:** Low 0–2, medium 3–4, high 5–6	**Scoring range:** 0–9 **Categories:** Low 0–3, medium 4–5, high 6–9	**Scoring range:** 0–6 **Categories:** Low 0–1, medium 2–3, high 4–6
**1) Meal pattern:** breakfast, lunch, dinner, and evening meal.	**1)** Consuming more **HM**^a^ **fruit puree** relative to CP^b^ fruit puree	**1) Fruits:** eating fruits more than 10.5 t/week	**1) Fruits:** eating fruits more than 7 t/week
**2) Nordic fruits:** apples, pears, plums, and strawberries.	**2)** Consuming more **HM dinners** relative to CP dinners	**2) Vegetables:** eating vegetables more than 5.5 t/week^c^	**2) Vegetables:** eating vegetables more than 5 t/week
**3) Root vegetables:** carrots, rutabaga, and various types of onions.	**3)** Consuming more **HM porridge** over CP porridge	**3) Peas and beans:** eating peas and beans more than 5 t/week	**3) Potatoes:** eating more potatoes over rice and pasta.
**4) Cabbages:** kale, cauliflower, broccoli, and Brussels sprouts.	**4)** Being exclusively breast-fed for at least 4 months (yes/no)	**4) Potatoes:** eating more potatoes over rice and pasta.	**4) Fish:** eating fish more than 2.12 t/week
**5) Potatoes:** eating more potatoes over rice and pasta	**5)** Any breastfeeding at 6 months (yes/no)	**5)** Eating more **HM porridge/baby cereal** over CP porridge/baby cereal	**5) Milk:** drinking more milk over fruit juice
**6) Whole grain breads:** eating more whole grain breads over refined breads	**6)** Drinking more **water** over sweetened beverages	**6) Fish:** eating fish more than 2.13 t/week	**6) Sweet beverages:** drinking sweetened beverages less than 2.5 t/week
**7) Oatmeal porridge:** frequency of eating oatmeal porridge		**7) Milk:** drinking more milk over fruit juice	
**8) Foods from the wild countryside:** game, fish, seafood and native berries.		**8) Water:** drinking more water over sweetened beverages	
**9) Milk:** drinking more milk over fruit juice		**9)** Eating more **HM dinners** over CP baby food	
**10) Water:** drinking more water over sweetened beverages			

Overview of the diet scores and the corresponding subscales assessing adherence to a healthy and potentially sustainable New Nordic Diet (NND) during pregnancy and child age 6 months, 18 months and 3 years. Food/drink items or words marked in bold reflects the scored item or valued behavior

^a^*HM* homemade

^b^*CP* commercially prepared

^c^*t/week* times a week

For each child score, dietary variables from the child questionnaires were selected to construct subscales based on the maternal NND subscales score. The questions assessing child diet were far less extensive and specific compared to the maternal FFQ, hence, the included dietary components differ to some degree from the maternal score and additionally differ between age-specific scores.

In the child dietary assessment, the mothers were mainly asked to respond to “How often does your child usually eat/drink the following” with response alternatives varying slightly between questionnaires. All responses were subsequently recoded to reflect a weekly consumption. We defined missing as having incomplete data on all food items that were included in the construction of each child diet score. If information was missing for some food items only, an assumption of null intake was made in accordance with recommendations by Cade et al. [28]. These items were recoded to 0 (never/seldom) to avoid losing all dietary information for respondents with incomplete data for a given item. For the maternal score, all missing items were null-imputed.

Further, the included food or drink items were added together in the respective subscales, either to yield a subscale measuring frequency of weekly consumption or to generate a relative measure of consumption of one food group compared to another. The subscales were mostly dichotomized by the median and coded to give either 0 or 1 point, where receiving 1 point acknowledged a healthier food choice or a frequency of consumption above the median of a healthy food item. Some subscales were scored according to responding “yes” or “no” to a question, where “yes” indicated the favourable health behaviour.

The sum of the subscales was further computed into continuous age-specific NND scores. Finally, each score was divided into low, medium, and high adherence groups with the intention to create as equally sized groups as possible. Where this was not possible, cut-offs were chosen to yield the low and high adherence groups as equally proportioned as possible. The rationale for categorizing participants into low, medium, and high adherence was to be able to compare high vs. lower adherence to the described dietary pattern. This was further used to explore associations between maternal and child diet and developmental outcomes in participating children.

### Child developmental outcomes

Child communication skills and motor development at 6 and 18 months, 3 and 5 years, respectively, were assessed with short forms of Ages and Stages Questionnaires (ASQ) and Child Development Inventory (CDI) [29]. ASQ is a parent-completed questionnaire tool that is used to identify potential developmental delay compared to age-peers, in need for further assessment. It is a widely used developmental screening-tool validated for use in Norwegian populations [30, 31] and has been applied in previous MoBa studies of prenatal exposure through maternal diet and child development [32, 33]. In MoBa, the ASQ at 5 years only covers language development. At this age, we used the CDI, completed by the parents in the 5-year questionnaire to assess motor skills and determine the child’s developmental level based on skill-assessment at given ages throughout the first 2 years in life and upwards to six and half years [34].

Outcome dimensions were defined according to the respective instrument manuals [29, 30, 35]. The outcome data were calculated as sum scores as the basis for the analysis, with a lower score indicating fewer milestones achieved by the child at the time of measure. We used simple imputation for participants with less than 50% of items missing on total scores. The missing items were recoded to mean of total score. Participants missing more than 50% of items in a score were excluded at that timepoint of measure. Sensitivity analysis between excluding missing values and imputed values were conducted and there was no significant change in results.

The items used to measure developmental skills changed across age as the child grew older, thus, the measures are not directly longitudinally comparable. The range of the outcome measures across the five timepoints of data collections also differ.

### Covariates

Covariates considered for inclusion in the models were baseline variables from questionnaires answered during pregnancy and at birth regarding maternal health and socioeconomic status identified as adjustment factors in previous studies investigating the relationship between diet and child development [32, 36]. The covariates included were parity, maternal age at delivery in four categories, maternal education, maternal pre-pregnancy body mass index (BMI) and marital status. Maternal symptoms of depression were additionally included as a covariate and was measured by a five-item short version of the Hopkins Symptom Checklist, psychometrically derived from the 25-item version [37]. Also included were child sex, gestational age (included in analysis with child NND scores), and age of the child when the questionnaire was answered.

### Statistical analysis

Linear regression and logistic regression analysis with robust standard errors were employed to compute crude and adjusted estimates of associations of maternal and age-specific child NND scores with measures of child development from 6 months to 5 years. Both methods were applied to establish a potential positive linear association between the NND scores and child development scores, and to examine whether scoring in the low NND score-categories was associated with developmental delay as expressed by scoring 2 standard deviations (SD) below or lower than the mean developmental score. The given cut-off allows for identification of the lowest scoring individuals within the sample, although the cut-off is not clinically validated for assessment of a specific developmental delay. The distribution of child development scores was highly left skewed with more than 90% of the children scoring within the 90th percentile range at each timepoint. After carefully considering the consequences of comparing exposure and outcome measurements which both had a different number of items and showed different range and SD at the different timepoints, we concluded that longitudinal analysis methods would not be suitable for this study. Instead, we performed cross-sectional analyses on the relationship between exposure and outcome at each timepoint and investigated potential trends and patterns in the results.

In the linear regression model, the percentage of maximum scoring for the developmental outcome and NND scores were computed to give more comparable results across timepoints. For the logistic regression models, the child development scores were dichotomized with 2SD below the mean as cut-off. Developmental score values at -2SD of the mean score or lower were assigned the value 1 (poor outcome), and the rest given the value 0. For these analyses, the values of the NND score categories (high/middle/low) were reversed with the low NND adherence group being assigned the value 2 and the high adherence was given the value 0 (reference group). More than 10% of the mothers participated more than once in the study and to correct for a possible impact of sibling covariance, we used robust cluster variance estimation in all analyses. The statistical programs, SPSS version 22 (SPSS, Inc., Chicago, IL, USA) and STATA/SE 16.1 were used for the analyses.

## Results

Basic maternal and child characteristics of the study sample are described in Supplementary Information (SI). In previous research, we have shown that mothers with high NND adherence during pregnancy were older, more educated and of higher parity than those with low NND adherence [18]. They were less likely to smoke, more likely to be normal weight and to exercise compared to women with low NND adherence.

Number of participants included in the analyses and properties of the NND scores and child development scores are listed in Table 2. The percentage of children scoring lower than the cut-off of 2SD below the mean developmental score ranged from 3.5% (18 months) to 5.2% (6 months).

**Table 2:** Description and distribution of the sample according to New Nordic Diet and child development scores

**New Nordic Diet score**	N	Mean NND score (SD)	Score range	Mean, % of total score	NND categories		
					Low %	Medium %	High%
Maternal pregnancy diet	83,800	4.9 (2.0)	0–10	49	26.1	35.2	38.7
Child diet 6 months	73,575	3.3 (1.3)	0–6	55	26.9	54.5	18.6
Child diet 18 months	62,601	4.2 (1.7)	0–9	47	33.4	43.2	23.3
Child diet 3 years	50,432	2.8 (1.4)	0–6	46	19.5	49.5	31.0
**Child development measure tools**^**a,b**^	N	Mean (SD)	Score range	Mean, % of total score	Below cut-off (<−2SD) N (%)		
Score 6 months	73,721	103.3 (8.7)	0–110	94	3845 (5.2)		
Score 18 months	63,065	117.0 (14.4)	0–130	90	2182 (3.5)		
Score 3 years	48,110	90.8 (10.1)	0–100	91	2200 (4.8)		
Score 5 years Language development	33,742	66.1 (6.8)	0–70	95	1290 (3.8)		
Score 5 years Motor development	35,027	11.0 (1.6)	0–12	92	1545 (4.1)		

Number of participants in the analyses and properties of New Nordic Diet (NND) scores (exposure) and child development outcomes at time of measure, such as number of participants, mean score, standard deviation (SD), and score range. Valid percent of participants in categories of low, medium and high adherence to the NND, and number and percentage of participants in the low score child development group (2SD below the mean)

^a^Short forms of Ages and Stages Questionnaire (ASQ) and Child Development Inventory (CDI)

^b^Developmental scores at 6 months, 18 months and 3 years are based on combining the communication and motor items

When examining the NND and child developmental scores as percentages of the total scores, we found an overall positive association between the NND scores and higher scoring on child development. For maternal pregnancy diet, the ${\hat{\beta}}$ 's and corresponding 95% CIs in the adjusted models ranged from $\hat{\beta}$: 0.012 (0.006, 0.017) (5 years, motor) to $\hat{\beta}$: 0.037 (0.033, 0.042) (developmental score at 18 months) (Table 3). For the child diet scores, ${\hat{\beta}}$ 's ranged from 0.007 (0.004, 0.009) (NND score at 6 months with developmental score at 6 months) to the strongest level of association observed in a cross-sectional manner at child age 18 months (0.045 [0.040, 0.050]) (Table 3). NND child diet scores at 18 months and 3 years have a relatively similar level of association to child development at the measured timepoints.

**Table 3:** Associations between the New Nordic Diet and child development examined as percentages of total score

Child development scores^**a**^		6 months	18 months	3 years	5 years, language	5 years, motor
NND score		$\hat{\beta}$ (95% CI)	$\hat{\beta}$ (95% CI)	$\hat{\beta}$ (95% CI)	$\hat{\beta}$ (95% CI)	$\hat{\beta}$ (95% CI)
**Maternal score**	Crude	0.020 (0.017, 0.023)	0.034 (0.029, 0.038)	0.027 (0.022, 0.031)	0.015 (0.010, 0.020)	0.028 (0.021, 0.035)
	Adjusted^b^	0.021 (0.018, 0.024)	0.037 (0.033, 0.042)	0.025 (0.020, 0.030)	0.012 (0.006, 0.017)	0.027 (0.020, 0.034)
**6 months**	Crude	0.010 (0.007, 0.013)	0.028 (0.024, 0.032)	0.018 (0.013, 0.022)	0.020 (0.015, 0.025)	0.015 (0.008, 0.021)
	Adjusted^c^	0.007 (0.004, 0.009)	0.021 (0.017, 0.025)	0.013 (0.009, 0.017)	0.013 (0.008, 0.018)	0.011 (0.005, 0.018)
**18 months**	Crude		0.063 (0.057, 0.069)	0.039 (0.033, 0.044)	0.025 (0.021, 0.029)	0.004 (0.003, 0.005)
	Adjusted		0.045 (0.040, 0.050)	0.036 (0.031, 0.042)	0.031 (0.025, 0.037)	0.030 (0.022, 0.038)
**3 years**	Crude			0.036 (0.032, 0.040)	0.016 (0.012, 0.019)	0.003 (0.003, 0.004)
	Adjusted			0.035 (0.031, 0.039)	0.019 (0.014, 0.024)	0.030 (0.023, 0.037)

Crude and adjusted linear regression of New Nordic Diet (NND) scores and child development scores, presented with $\hat{\beta}$ and 95% confidence intervals (CI)

^a^Short forms of Ages and Stages Questionnaire (ASQ) and Child Development Inventory (CDI)

^b^Adjusted for maternal age, marital status, maternal education, maternal BMI, maternal depression, parity, siblings included in the study population and child age at completion of questionnaire

^c^Gestational age was additionally adjusted for in analysis of child diet

**Fig. 2:**
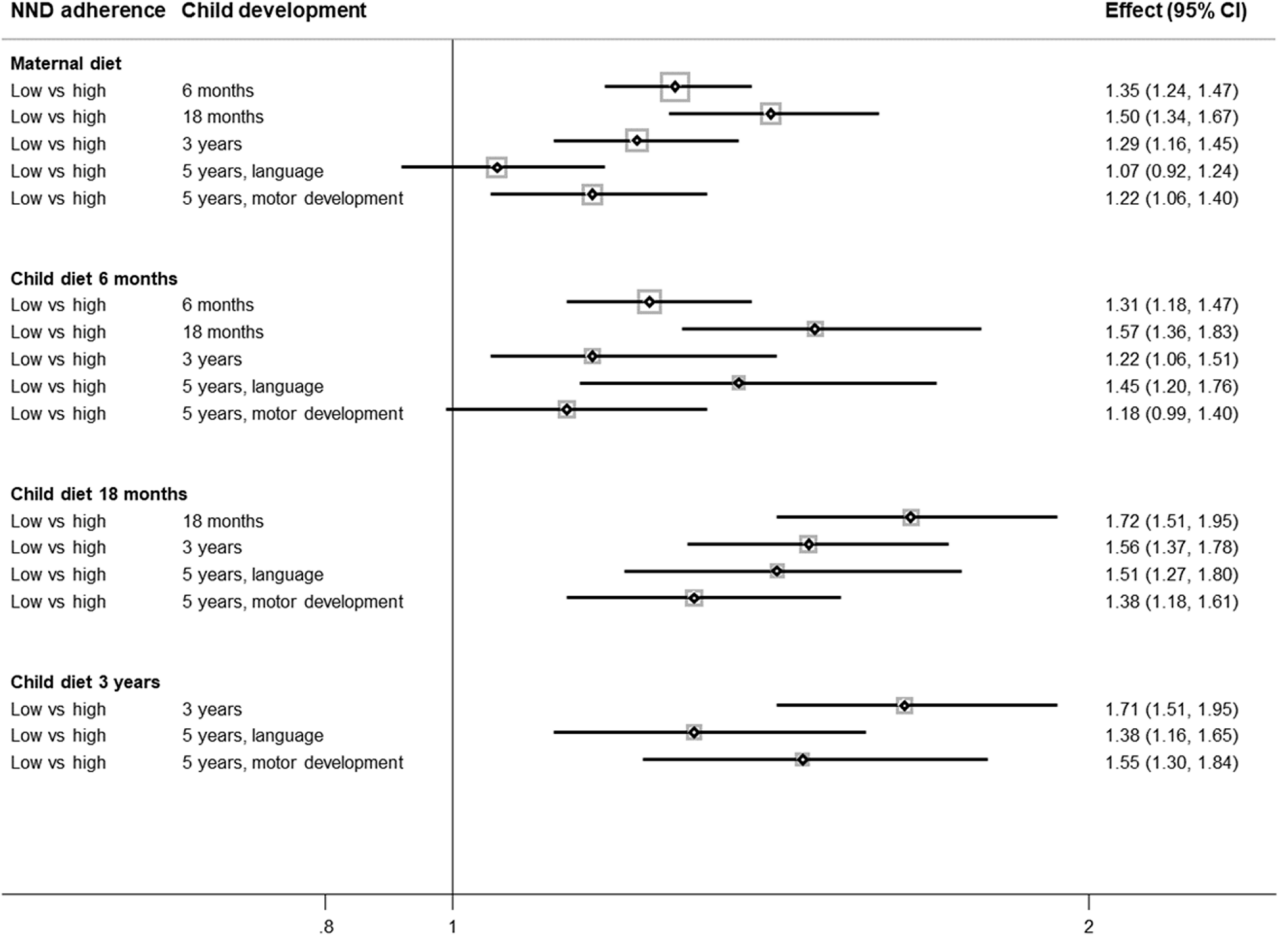
Adjusted odds ratios and 95% confidence intervals (CI) for delayed development with low vs. high adherence to the New Nordic Diet (NND). Delayed development is defined by scoring 2 standard deviations below the mean on short forms of the Ages and Stages Questionnaire (ASQ) and the Child Development Inventory (CDI; 5 years, motor development)

**Fig. 3:**
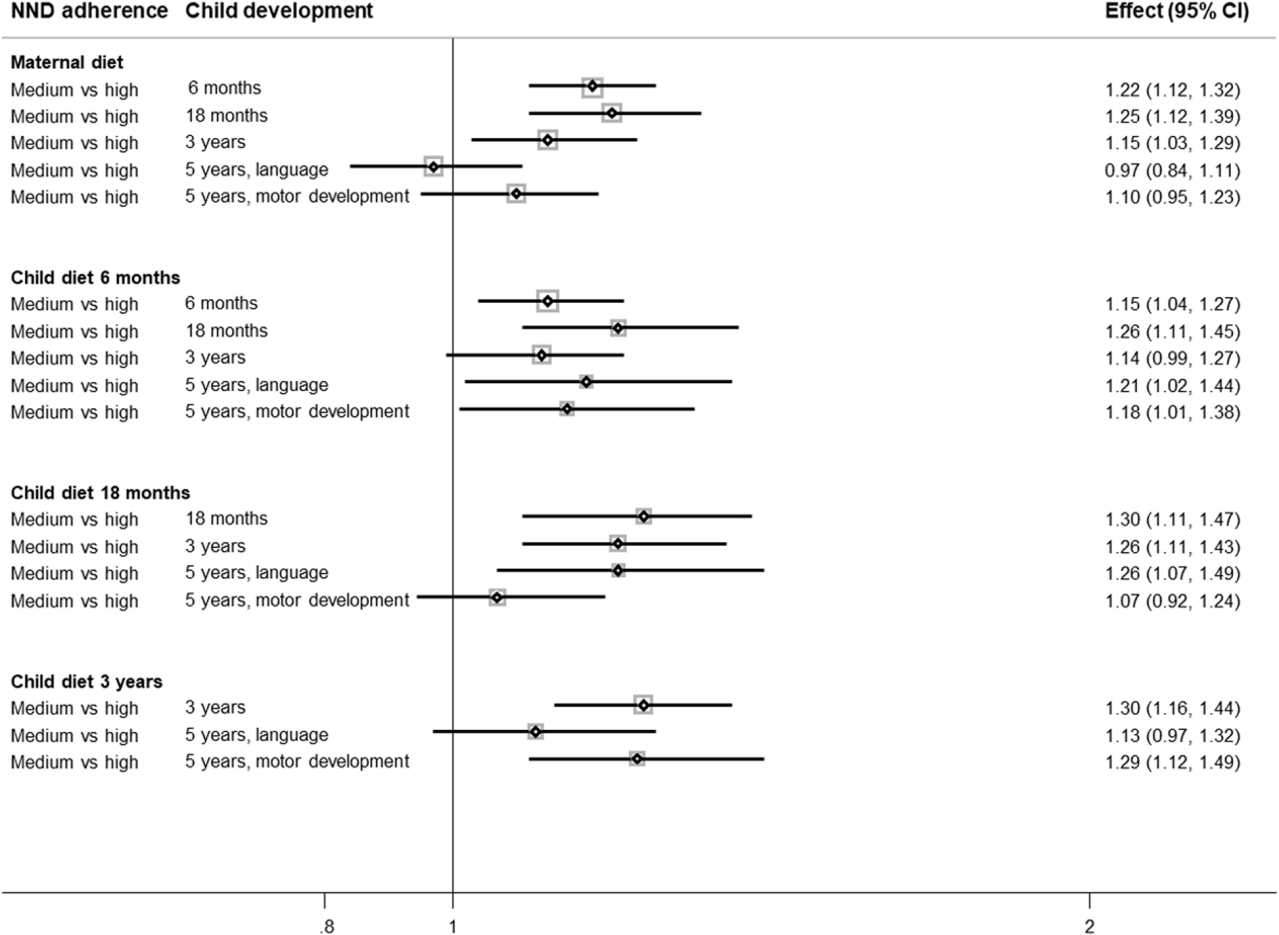
Adjusted odds ratios and 95% confidence intervals (CI) for delayed development with medium vs. high adherence to the New Nordic Diet (NND). Delayed development is defined by scoring 2 standard deviations below the mean on short forms of the Ages and Stages Questionnaire (ASQ) and the Child Development Inventory (CDI; 5 years, motor development)

We investigated the odds of scoring low on age-specific developmental measures with low or medium vs. high NND adherence category at any timepoint (Table 4, Figs. 2 and 3).

**Table 4:** Associations between categorized age-specific New Nordic Diet scores and odds of delayed development

Child development scores															
	6 months			18 months			3 years			5 years, language			5 years, motor		
NND adherence categories	Low	Medium	High (ref)	Low	Medium	High (ref)	Low	Medium	High (ref)	Low	Medium	High (ref)	Low	Medium	High (ref)
**Maternal pregnancy NND score, OR (95% CI)**															
Crude	1.33* (1.22, 1.45)	1.21* (1.12,1.31)	1	1.45* (1.30, 1.61)	1.24* (1.12, 1.37)	1	1.31* (1.17, 1.45)	1.16* (1.05, 1,28)	1	1.14 (0.99, 1.31)	1.00 (0.88, 1.14)	1	1.23* (1.08, 1.41)	1.13 (0.99, 1.28)	1
Adjusted^a^	1.35* (1.24, 1.47)	1.22* (1.12,1.32)	1	1.50* (1.34, 1.67)	1.25* (1.12, 1.39)	1	1.29* (1.16, 1.45)	1.15* (1.03, 1.29)	1	1.07 (0.92, 1.24)	0.97 (0.84, 1.11)	1	1.22* (1.06, 1.40)	1.10 (0.95, 1.23)	1
**Child NND score 6 months, OR (95% CI)**															
Crude	1.54* (1.39, 1.71)	1.31* (1.19,1.44)	1	1.79* (1.55, 2.06)	1.39* (1.21, 1.58)	1	1.40* (1.22, 1.60)	1.21* (1.07, 1.37)	1	1.65* (1.39, 1.97)	1.22* (1.04, 1.44)	1	1.22* (1.03, 1.44)	1.18* (1.02, 1.37)	1
Adjusted^b^	1.31* (1.18, 1.47)	1.15* (1.04,1.27)	1	1.57* (1.36, 1.83)	1.26* (1.10, 1.45)	1	1.22* (1.06, 1.51)	1.14 (0.99, 1.27)	1	1.45* (1.20, 1,76)	1.21* (1.02, 1.44)	1	1.18 (0.99, 1.40)	1.18* (1.01, 1.38)	1
**Child NND score 18 months, OR (95% CI)**															
Crude				1.76* (1.56, 2.00)	1.31* (1.16, 1,48)	1	1.61* (1.42, 1.83)	1.27* (1.12, 1.44)	1	1.68* (1.43, 1.99)	1.32* (1.12, 1.55)	1	1.38* (1.19, 1.60)	1.06 (0.91, 1.22)	1
Adjusted				1.72* (1.51, 1.95)	1.30* (1.10, 1.47)	1	1.56* (1.37, 1.78)	1.26* (1.11, 1.43)	1	1.51* (1.27, 1.80)	1.26* (1.07, 1.49)	1	1.38* (1.18, 1.61)	1.07 (0.92, 1.24)	1
**Child NND score 3 years, OR (95% CI)**															
Crude							1.77* (1.56, 2.00)	1.33* (1.19, 1.48)	1	1.50* (1.27, 1.79)	1.20* (1.04, 1.39)	1	1.50* (1.28, 1.77)	1.24* (1.08, 1.42)	1
Adjusted							1.71* (1.51, 1.95)	1.30* (1.16, 1.44)	1	1.38* (1.16, 1.65)	1.13 (0.97, 1.32)	1	1.55* (1.30, 1.84)	1.29* (1.12, 1.49)	1

Odds ratios (OR) and 95% confidence intervals (CI) of association between New Nordic Diet (NND) adherence categories (low, medium, high) and delayed child development (scoring 2 standard deviations below the mean)

^a^Adjusted for maternal age, civil status, maternal education, maternal BMI, maternal depression, parity, siblings included in the study population and child age at completion of questionnaire

^b^Gestational age was additionally adjusted for in analysis of child diet

^*^p-value < 0.05

Compared with high NND adherence, low and medium adherence categories were associated with higher odds of delayed development at almost all measure points (OR range within significant values in adjusted models: low vs. high: OR; 1.22 [1.06, 1.51] to 1.72 [1.51, 1.95] and medium vs. high: OR; 1.15 [1.04, 1,27] to 1.30 [1.1, 1,47]). We see sustained higher odds of delayed development with low vs. high NND categories, with an exception for the maternal NND score with language outcome at 5 years (OR; 1.07 [0.92, 1.24]) and child NND score at 6 months with motor developmental outcomes (OR 1.18 [0.99, 1.40]) reported at 5 years. Nearly the same tendency is seen for medium vs. high NND adherence.

## Discussion

In this study, we report relatively consistent and robust findings that a healthy diet in early life is positively associated with measures of child development. The robustness of the results was confirmed by analyzing the data both as continuous and binary variables. Despite the crude way of scoring healthy food intake in the diet score development, we found an overall positive association between higher NND score and scoring better on child development measures, including communication and motoric assessment in this cohort. We also found that being in the low NND adherence group yielded higher odds of delayed development (developmental scores <−2SD) compared to high NND adherence in a dose-response fashion. These associations were found at nearly all measured timepoints of dietary exposure from pregnancy to 6 months, 18 months and 3 years with development at 6 and 18 months and 3 and 5 years. The findings are in line with other European studies which have found that higher adherence to healthy dietary patterns is associated with improved school performance [24] and higher childhood IQ [38]. We did not observe an association at 5 years (language) with high vs. low or medium maternal NND adherence, which could imply that other factors than maternal diet during pregnancy may be of more importance for language development by this age.

Previous studies in MoBa have shown that maternal diet quality described by various measures of a healthy diet, is associated with a decrease in ADHD symptoms and diagnosis at 8 years [39], and that higher maternal intake of unhealthy foods during pregnancy predict emotional problems among children at the age of five [40]. However, our study is, to our knowledge, one of few that has explored associations between both maternal and child dietary patterns and child communication and motor development at multiple timepoints. Showing robust associations with measures of diet quality over time from the fetal life in the womb until 5 years of age, reinforces the importance of overall diet quality, and probably specific nutrients, on child communication and motor development. This is corroborated by current knowledge that healthy dietary patterns may promote child neurocognitive development through changes in cellular processes, neuroplasticity, or epigenetic mechanisms, as stated by Tandon et al. [4, 5].

It should be noted that the observed associations showing higher development scores with healthier diet are found in a presumably healthy Norwegian population with no lack of access to healthy food, most parents able to afford a healthy diet for their child, and with dietary guidelines easily accessible. This finding shows that a suboptimal diet can potentially provide limitations on a child’s cognitive development and is not confined only to developing countries [6, 41], but also holds for developed countries. A nation’s prosperity and health rely on the healthy development of their younger generations. Therefore, a larger focus on how to provide a healthy diet, is important in early childcare. Health authorities should take note that a healthy diet is important not only for short- and long-term health, but presumably for early cognitive and motor development as well. The diet scores applied in these analyses were constructed to capture potentially sustainable aspects of diet. Given the recent increased attention on the necessity of having more sustainable diets [12], the beneficial associations of higher diet scoring with aspects of child development have additional benefits that should also be conveyed to the public.

### Strengths and limitations

The strengths of the present study include the large sample size and prospective analyses linking diet and cognitive development at multiple timepoints from pregnancy throughout early childhood. Additionally, several potential confounders known to be associated with maternal and child diet and cognitive development were accounted for in the adjusted models. Nonetheless, there are several limitations to consider. Although the child diet scores were developed on the rationale of capturing a healthy, local, and potentially sustainable Nordic diet, the dietary assessment in the child questionnaires were not as comprehensive as in the maternal FFQ. This may have limited the child scores’ ability to truly reflect a sustainable diet with Nordic characteristics as intended. This is discussed thoroughly elsewhere [24].

The NND scores are constructed to capture adherence to a healthy Nordic diet by measuring frequency of intake of some key healthy food items in the diet. As the diet scores do not measure unhealthy food items directly, a low score is characterized by having less of the healthy food items and therefore primarily reflects the consequence of not having these healthy food items in the diet. Moreover, the method chosen to define cut-offs for the NND categories may have contributed to misclassifications due to the arbitrary approach. On the other hand, the normal distribution of the scores was carefully interpreted when deciding the cut-off values. Furthermore, the use of median values as cut-off in most subscales of the age-specific diet scores were also data-driven based on dietary intake in the MoBa cohort, which may not be representative for the rest of the population. The mothers participating in MoBa were older (> 25), more often cohabitating and more frequent users of multivitamin and folic acid supplements compared to non-participating mothers in Norway [42]. Smokers and mothers with more than two previous births were underrepresented. Hence, self-selection bias and residual confounding cannot be ruled out. Also, with self/parent-reported dietary data, the possibility of misclassification to the score categories due to social desirability bias cannot be excluded.

The shortened ASQ subscales used in the MoBa holds another potential limitation. The MoBa questionnaires covered a multitude of developmental and mental health measures and provided limited space. The child’s age at completion of the questionnaires varied and the selected items would therefore be adequate for somewhat younger/older aged children as well. To account for this, the age at completion of the questionnaire was used as a covariate in the analyses.

The ASQ is regarded as an effective diagnostic tool of developmental delay and/or deviance [30, 35]. Yet, the shortened instrument used in the current study is limited to ascertain associations between differential diet quality and severely poor scoring (<−2SD) or higher scoring on ASQ. The findings would translate to whether the child is reaching developmental milestones slower or faster than expected for the child’s given age. A low score (<−2SD) on the shorter ASQ is not necessarily reflecting a clinically defined developmental delay impacting the child’s daily functioning. For such a conclusion to be made, a more complete assessment would be necessary with broader developmental scales and parent-completed questionnaires on child impairment.

Furthermore, both exposure and outcome variables examined in this study, differed from one-another at each timepoint, preventing us from assessing the relationship between a healthy and potentially sustainable Nordic diet and child development with repeated measures methods. The heterogeneity within the NND scores and the shortened ASQ limits the possibility to compare effect sizes across age-specific analyses, as they capture the NND and child development differently at each timepoint.

Finally, as the current study has data derived from an observational study, any causal interpretation cannot be ascertained. Still, it should be noted that although the effect size for most associations were small, they were remarkably consistent and perhaps likely to be of relevance in a public health perspective.

## Conclusion

We found a robust association between a healthy and potentially sustainable diet early in life and child communication and motoric development in the Norwegian Mother, Father and Child Cohort Study. This association was found at several timepoints, from maternal diet during pregnancy to child diet at age 3 years. Our results highlight diet quality as a prerequisite for optimal development and reaching your potential, which is also relevant in developed countries, such as Norway. A higher focus on the relevance of diet for communication and motoric development beyond physical health and growth seems warranted.

## Supplementary Information

**Additional file 1: Supplementary Information 1.** Summary statistics for maternal and child characteristics.

## Data Availability

The consent given by the participants does not open for storage of data on an individual level in repositories or journals. Researchers who want access to data sets for replication should submit an application to datatilgang@fhi.no. Access to data sets requires approval from The Regional Committee for Medical and Health Research Ethics in Norway and an agreement with MoBa.

## Abbreviations

ASQ: Ages and Stages Questionnaire
BMI: Body mass index
CDI: Child Development Inventory
CI: Confidence interval
FFQ: Food frequency questionnaire
MBRN: Medical Birth Registry of Norway
MoBa: The Norwegian Mother, Father and Child Cohort Study
NND: New Nordic Diet
OR: Odds ratio
SD: Standard deviation
