# Supplementary material for: Adherence to a healthy and potentially sustainable Nordic diet is associated with child development in The Norwegian Mother, Father and Child Cohort Study (MoBa)
Source: Nutr J. 2022 Jul 18;21:46. doi: 10.1186/s12937-022-00799-5 (PMC9290263; doi:10.1186/s12937-022-00799-5)
Supplement: Supplementary file 1 — Additional file 1: Supplementary Information 1. Summary statistics for maternal and child characteristics. [file 12937_2022_799_MOESM1_ESM.docx]

# **Adherence to a healthy and potentially sustainable Nordic diet is associated with child development in the Norwegian Mother, Father and Child Cohort Study (MoBa)** *Nutrition Journal*

Kristine Vejrup, (1,2)

*Neha Agnihotri (1)

Elling Bere (3,4)

Synnve Schjølberg (5)

Marissa LeBlanc, (1,6)

Elisabet Rudjord Hillesund (1)

Nina Cecilie Øverby (1)

(1), Department of Nutrition and Public Health, Center for Lifecourse Nutrition, University of Agder, Kristiansand, Norway
(2) Norwegian Armed Forces Medical Services, Sessvollmoen, Norway
(3) Department of Sport Science and Physical Education, University of Agder, Kristiansand, Norway
(4) Department of Health and Inequalities & Centre for Evaluation of Public Health Measures, Norwegian Institute of Public Health, Oslo, Norway
(5) Department of Child Health and Development, Norwegian Institute of Public Health, Oslo, Norway
(6) Oslo University Hospital, Oslo Centre for Biostatistics and Epidemiology, Oslo, Norway

*Email address of corresponding author:

neha.agnihotri@uia.no

**Supplementary Information 1.** Summary statistics for maternal and child characteristics.

|  |  | **N (%)** | |
| --- | --- | --- | --- |
| Maternal age at delivery | <25 y | 8814 | (10.5) |
|  | 25-29 y | 27,594 | (32.9) |
|  | 30-34 y | 32,594 | (38.9) |
|  | 35+ y | 14,798 | (17.7) |
| Pre-pregnancy BMI (kg/m²) | 18,5-24,9 | 53,527 | (63.9) |
|  | <18,5 | 2443 | (2.9) |
|  | 25-29,9 | 17,844 | (21.3) |
|  | 30+ | 7838 | (9.4) |
|  | Missing | 2148 | (2.6) |
| Educational level | =<12 | 25,168 | (30.0) |
|  | 13 - 16 y | 35,179 | (42.0) |
|  | 17+ y | 21,744 | (25.9) |
|  | Other/missing | 1709 | (2.0) |
| Smoking during pregnancy | No | 76,990 | (91.9) |
|  | Yes | 6202 | (7.4) |
|  | Missing | 608 | (0.7) |
| Alcohol use during pregnancy | Never/rarely | 70,791 | (84.5) |
|  | 0,5 - 3 times/week | 1926 | (2.3) |
|  | >3 times/week | 20 | (0) |
|  | Missing | 11,063 | (13.2) |
| Breastfeeding | No | 11,064 | (13.2) |
|  | Yes | 72,736 | (86.8) |
|  |  |  |  |
| Parity | Nulliparous | 38,254 | (45.6) |
|  | Multiparous | 45,546 | (54.4) |
| Child gender | Boy | 42,906 | (51.2) |
|  | Girl | 40,833 | (48.7) |
|  | Missing | 61 | (0.1) |
